# PRIMARY ABANDON-OF-THE-SAC (PAS) TECHNIQUE: PRELIMINARY RESULTS OF A NOVEL MINIMALLY INVASIVE APPROACH FOR INGUINOSCROTAL HERNIA REPAIR

**DOI:** 10.1590/0102-672020200002e1519

**Published:** 2020-11-20

**Authors:** Alexander Charles MORRELL, Andre Luiz Gioia MORRELL, Flavio MALCHER, Allan Gioia MORRELL, Alexander Charles MORRELL-JUNIOR

**Affiliations:** 1 Morrell Institute, Minimally Invasive and Robotic Digestive System Surgery, São Paulo, SP, Brazil; 2 Brazilian Israeli Beneficent Society Albert Einstein, Digestive System Surgery, São Paulo, SP, Brazil; 3 Network D’Or São Luiz, Surgery of the Digestive System and Robotics, São Paulo, SP, Brazil; 4 Montefiore University Hospital, Department of Surgery, New York, NY, United States

**Keywords:** Hernia, inguinal, Seroma, Laparoscopy, Hernia, Hérnia inguinal, Seroma, Laparoscopia, Hérnia

## Abstract

**Background:**

Laparoscopic best approach of repairing inguinoscrotal hernias are still debatable. Incorrect handling of the distal sac can possibly result in damage to cord structures and negative postoperative outcomes as ischemic orquitis or inguinal neuralgia.

**Aim:**

To describe a new technique for a minimally invasive approach to inguinoscrotal hernias and to analyze the preliminary results of patients undergoing the procedure.

**Methods:**

A review of a prospectively maintained database was conducted in patients who underwent minimally invasive repair using the “primary abandon-of-the-sac” (PAS) technique for inguinoscrotal hernias. Patient´s demographics, as well as intraoperative variables and postoperative outcomes were also analyzed.

**Results:**

Twenty-six male were submitted to this modified procedure. Mean age of the case series was 53.8 years (range 34-77) and body mass index was 26.8 kg/m^2^ (range 20.8-34.2). There were no intraoperative complications or conversion. Average length of stay was one day. No surgical site infections, pseudo hydrocele or neuralgia were reported after the procedure and two patients presented seroma. No inguinal hernia recurrence was verified during the mean 21.4 months of follow up.

**Conclusion:**

The described technique is safe, feasible and reproducible, with good postoperative results.

## RESUMO

**Racional:**

A melhor maneira laparoscópica do reparo de hérnias inguinoescrotais permanece ainda aberta a discussão. O manuseio incorreto do saco herniário pode resultar em danos às estruturas do cordão e resultados pós-operatórios indesejados, como orquite isquêmica ou neuralgia inguinal.

**Objetivo:**

Descrever uma nova técnica de abordagem minimamente invasiva das hérnias inguinoescrotais e analisar os resultados preliminares de pacientes submetidos ao procedimento.

**Métodos:**

Foi realizada na série de casos a análise retrospectiva de um banco de dados mantido prospectivamente em pacientes submetidos a reparo minimamente invasivo usando a técnica “abandono primário do saco” (PAS) para hérnias inguinoescrotais. Dados demográficos dos pacientes, bem como variáveis intraoperatórias e resultados pós-operatórios são descritos.

**Resultados:**

Vinte e seis homens foram submetidos ao procedimento. A idade média foi de 53,8 anos e o índice de massa corpórea de 26,8 kg/m^2^. Não houve complicações intraoperatórias ou conversão para operação aberta. A duração média da estadia hospitalar foi de um dia. Não foram relatadas infecções de sítio cirúrgico, hematomas ou neuralgia após o procedimento e a presença de seroma ocorreu em dois pacientes. Nenhuma recorrência de hérnia inguinal foi verificada durante média de 21,4 meses.

**Conclusão:**

A técnica descrita é segura e replicável, apresentando bons resultados pós-operatórios.

## INTRODUCTION

The success of TAPP (transabdominal preperitoneal) or TEP (totally extraperitoneal) approach for inguinal hernias is evident. Laparoscopic hernioplasty have resulted in early recovery to normal activities and a lower incidence of wound infection. However, the best approach in repairing large inguinoscrotal hernias and the optimal management of the distal sac and its risks are still debated. Visceral or cord structures damage, seroma, hematoma, as well as ischemic orchitis are not negligible when opting for a complete dissection of the hernia sac which may extend deep into the scrotum. Ferzly and Kiel^7^ first described an extraperitoneal approach for repair of large inguinoscrotal hernias in 17 patients having acceptable results in 1996, with no recurrence. In 2000, Liebl et al^12^ reported a transabdominal preperitoneal approach for discussion on the efficiency and complications of laparoscopic treatment of inguinoscrotal hernias. Since then, guidelines for laparoscopic and endoscopic treatment of inguinal hernia described that minimally invasive approaches are possible therapeutic options in inguinoscrotal hernias^2^. The absence of large scale comparative study is likely due to the relatively low number of cases.

Trakarnsagna et al^18^ suggested a giant inguinoscrotal hernia classification, arranging the cases in three types of stratification based on scrotum length. According to its classification, the more distal extension the sac is below mid inner thigh, the more unlikely a hernioplasty with forced reduction procedure is safe due to intra abdominal pressure control. In the type I hernia, a forced reduction of content with hernioplasty is feasible.

In this article we describe a novel minimally invasive TAPP approach for treatment of inguinoscrotal hernias pursuing a reproducible and safe manner to overcome the difficulties of the distal sac management. 

## METHODS

### Study design

**FIGURE 1:**
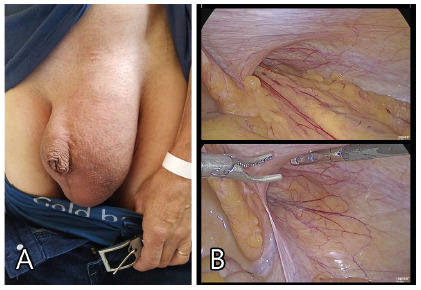
Views of left inguinoscrotal hernia sac: A) external; B) laparoscopic

A review of a prospectively maintained database was conducted from January 2014 to February 2019 in patients who underwent PAS (Primary Abandon-of-the-Sac) technique minimally invasive inguinoscrotal hernia repair. A total of 26 men were identified. To all patients with indication to inguinoscrotal hernia repair were offered the primary abandon-of-the-sac minimally invasive repair unless they were not considered fit for a general anesthesia procedure. Was considered inguinoscrotal hernia to be large when the distal sac extended deeply into the scrotum (Figure 1A). No large type II or III giant hernia according to Trakarnsagna et al^18^ classification were included. 

This technique is based on the manner of the peritoneal flap approach and hernia sac management. A step-by-step content for a systematic approach including patient´s demographics, hernias characteristics, perioperative variables and early postoperative outcomes were described. All patients were seen in follow-up clinic between 8 and 14 postoperative day by the same surgical group who maintained frequent clinical appointments and kept a database recording finding of postoperative seroma, hematoma, ischemic orchitis and recurrence (detected by physical examination of the groin) and pain (based on personal questioning and numerical rating scale). The study Institutional Review Board approval was obtained by the hospital ethical committee. 

### Patient preparation and ports placement

Under general anesthesia, the patient is positioned supine with arms close to the trunk. A Foley catheter is inserted and antibiotic prophylaxis is routinely used, consisting of administration of 1 g intravenous cefazolin in anesthetic induction. Whenever possible, forced hernia reduction is performed. The surgeon is placed lateral to the patient, contralateral to the defect to be repaired. A small infraumbilical incision is made; pneumoperitoneum is achieved by a Veress needle puncture and carbon dioxide insufflation. A 10 mm camera port is inserted into the abdominal cavity. Two 5 mm ports are placed either side lateral to the umbilicus, slightly above or below umbilical imaginary line**.**

### Primary abandon-of-the-sac and technical considerations

**FIGURE 2:**
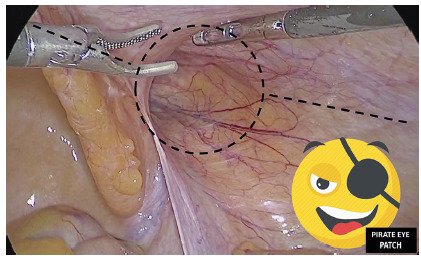
The “pirate-eye-patch” peritoneal flap dissection draft, with the primary abandon-of-the-sac approach

**FIGURE 3:**
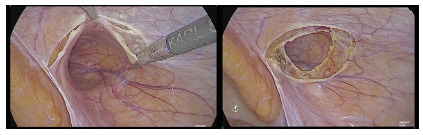
Peritoneal flap dissection bordering the hernia defect anterior and posteriorly, leaving both dissected planes in an ellipsoid or circular shape

**FIGURE 4:**
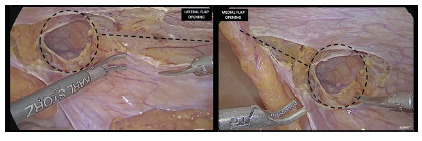
Both medially and laterally extension of the peritoneal flap dissection: medial limit - medial umbilical ligament; lateral limit - approximating the anterosuperior iliac spine

**FIGURE 5:**
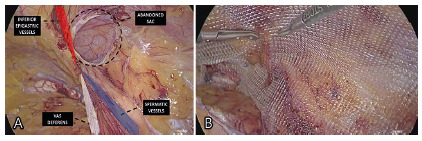
A) View of the right myopectineal orifice after complete dissection, with the abandoned sac circled and anatomical landmarks enhanced; B) final view of the implanted mesh

Once all ports are correctly positioned, a more accurate inspection of the hernia defect is done, and the intraperitoneal hernia sac content is reduced completely (Figure 1B). Based on a non-complete distal sac dissection, in a “pirate eye patch” (Figure 2) shape the abandon-of-the-sac approach is performed by a peritoneal flap dissected bordering the hernia defect anterior and posteriorly, leaving the distal hernia sac into the inguinal canal and scrotum (Figure 3). After creating an ellipsoid or circular shape, dissection to both medial and lateral direction is achieved, reaching the medial umbilical ligament or further and beyond the anterosuperior iliac spine respectively (Figure 4). This created flap develops a substantial extraperitoneal surgical field in a simple and prompt manner, assuring a perfect established critical view of the myopectineal orifice (Figure 5). By abandoning the circular-shape distal hernia sac inside the inguinal canal there is no necessity of dissection of the cord structures deep inside the inguinal canal from the peritoneum herniated. After a complete myopectineal orifice exposition, and its view achieved, the nine steps described by Jorge Daes^6^ are performed. They are summarized: 1) identify and dissect the pubic tubercle across the midline and Cooper ligament; 2) rule out a direct hernia; 3) dissect at least 2 cm between Cooper ligament and the bladder; 4) dissect between Cooper ligament and the iliac vein to identify the femoral orifice and rule out a femoral hernia; 5) dissect the indirect sac; 6) identify and reduce cord lipomas; 7) dissect peritoneum lateral to the cord’s elements laterally beyond the anterosuperior iliac spine; 8) perform the dissection, provide mesh coverage, and ensure that mesh and mechanical fixation are placed well; 9) place the mesh only when items 1 to 8 are completed and hemostasis has been verified. 

When treating associated inguinal direct hernia defects, we prefer not to suture or approximate the edges due to risk of neural injury. Mesh is placed covering completely the myopectineal orifice without bending edges and its fixation is performed using tacks: in the triangular sheet of the linea alba fibers that attaches posteriorly to the crest of the pubis inferiorly, rectal medial edge superomedially and abdominal wall laterally. After adequate mesh placement the peritoneal flap is sutured using a 3-0 barbed suture. A decrease of the pneumoperitoneum pressure to 8-10 mmHg is helpful when approximating the peritoneal edges. Neither surgical drain or fibrin sealant application is performed. Ports are retracted and the umbilical port is closed using 0 Vicryl.

## RESULTS

Twenty-six patients with inguinoscrotal hernia were submitted to the reported technique. The mean age was 53.8 years (34-77) with a mean BMI of 26.8 kg/m^2^ (20.8-34.2). Patient´s demographic and perioperative variables are in Table 1. None of the patients were converted to open technique. Mean procedure time was 48.6 min (40.9-142). There was no intraoperative complication, no 30-days emergency department readmission, neither 30-days mortality. Mean hospital length of stay was one day. Only two patients presented with seroma (7.6%). The first one referred sort of bulging in the inguinal region on the 11^th^ postoperative day returning for the scheduled appointment, being detected by physical examination and confirmed by ultrasonography. Treatment was achieved by ultrasonography guided percutaneous drainage, with complete resolution and no recurrence within 18 months of follow-up. The second patient presented on the 8^th^ postoperative day at the clinical appointment with no complains but during the physical exam, a seroma was detected and confirmed by ultrasonography. Treatment was expectant without puncture of the area and subsequent CT scan showed no abnormality in the area within six months of follow-up. No surgical site infection, hematoma or ischemic orquitis occurred. By actively questioning, neither neuralgia or testicle pain related to surgery were mentioned. There were no major complications or hernia recurrence. The mean follow-up period was 21.4 months (6-45).

**TABLE 1:** Patient´s demographic and perioperative variables

Patients	Value (n=26); n (%)
Gender male/female	26/0 (100/0%)
Age (years)	Mean 53.8 Range (34-77)
BMI (kg/m2)	26.8 Range (20.8-34.2)
ASA score - I/II - III - IV/V	26 (100%) 24 (92.4%) 2 (7.6%) 0 (0%)
Comorbidities - Hipertension - Diabetes - Obesity - COPD - Coronary artery disease	14 (53.8%) 8 (30.6%) 4 (15.3%) 4 (15.3%) 1 (3.8%) 1 (3.8%)
Procedure time (min)	Mean 48.6 Range (40.9-142)
Postoperative complications - Seroma - Hematoma - Surgical site infection - Ischemic orchitis - Pseudo hydrocele - Neuralgia - Recurrence	2/26 (7.6%) 2 (7.6%) 0 (0%) 0 (0%) 0 (0%) 0 (0%) 0 (0%) 0 (0%)
Length of hospital stay (days)	Mean 1.05 Range (1-2)
Postoperative follow-up (months)	Mean 21.4 Range (6-45)

## DISCUSSION

Hernias high prevalence and its impact on quality of life still a major healthcare issue. Inguinal hernia repair is the most frequent operation in general surgery worldwide. The use of laparoscopy had an important appeal for having lesser wound complications, with less tissue damage and providing better and faster recovery^15^. However, when treating inguinoscrotal hernias, there’s still no consensus on what’s the best surgical approach. Both TAPP and TEP minimally invasive procedures have been considered possible therapeutic options in inguinoscrotal hernias however literature is scant^2^.

Seroma is the most common postoperative complication after laparoscopic inguinal hernia repair^13^. Previous meta-analysis has showed higher seroma rates in laparoscopic hernia repair compared with conventional techniques (3.6-4.4 vs 0.5-1.2%)^3^ ^,^ ^17^. Our case series report an overall seroma rate of 7.6%, consistent with previous reports for TAPP of 8.0%^10^. Liebl et al^12^ described 10.5% of seroma occurrence in laparoscopic standard TAPP repair of scrotal hernias, with higher rates compared the normal inguinal procedures (4.4%). Interestingly, comparing a complete reduction of the hernia sac or performing its transection when necessary showed no difference in the seroma rates (10.5 vs 10.4%). However, the complete reduction of the hernia sac group had higher hydrocele as well as testicular atrophy and cutaneous sensory deficit rates. Therefore, it’s important to state that opting for an abandon of the hernia sac technique does not conjecture higher rates of seroma or pseudo hydrocele at all, on the contrary, reduces the operation time and possibly avoid complications such as chronic pain^14^. Among variables regarding reducing seroma formation, only the use of surgical drains met the criteria to be significantly effective^3^.

Bittner^4^ reported an analysis of 440 scrotal hernias in a large single-center series of 8.050 TAPP repairs. The overall recurrence for the series was 0.7% but 2.7% for scrotal hernias. In terms of hernia recurrence, its presentation may be attributed to a variety of causes. Incorrect space dissection for mesh placement, an excessively small mesh usage without adequate defect overlap, surgeons learning curve are possibly examples. However, when dealing with large hernia defects, insufficient mesh fixation or an over flexure of the prosthesis may cause its slippery into the defect^11^. Therefore, according to Hollinsky et al^8^, meshes with greater flexural stiffness or well-fixed lightweight meshes with adequate overlap are advised for laparoscopic treatment of large inguinal hernias. Our preference is to use at least a 15x10cm polypropylene high-density mesh piece, possibly larger according to necessity, added to adequate tack fixation.

In our opinion, a transabdominal preperitoneal approach for inguinoscrotal hernia repair shows many advantages. This minimally invasive approach allows direct observation of hernia contents from the intra-abdominal space added to a broad posterior view of the hernia defect size and the myopectineal orifice. Moreover, the PAS technique links the benefits from the laparoscopic transabdominal technique to a prompt and cord structures preserving approach independent of the inguinoscrotal hernia presentation.

Robotic surgery has gained popularity with improved dexterity, three-dimensional view and possibly more accurate and safe procedures, besides improvement of surgeon ergonomics. Its benefits in comparison to laparoscopy approach have already been well characterized in urology and gynecology surgical field^9^. Bariatric procedures have also shown its feasibility and good results^1^. One of the biggest concerns over performing robotic-assisted surgery is cost, however a recent report regarding robotic and laparoscopic procedures for inguinal hernia repair shows no significant difference. The robotic TAPP inguinal hernia repair had longer operative times but patients spent a shorter amount of time in recovery and noted less pain than patients who had laparoscopic TAPP inguinal hernia repair^19^ ^,^ ^16^. Even single port inguinal hernia procedures have also been described in the robotic platform^5^. Although literature still scarce, we believe robotic surgery may encourage better outcomes in inguinoscrotal hernias, overcoming laparoscopic limitations in more complex cases.

This described technique to deal with the distal sac in large inguinoscrotal hernias has been used for more than 15 years by our surgical group, however, no detailed and consistent data neither objective standardized postoperative evaluation had been done since then. A consistent group reorganization regarding data collection and standardized surgical procedures and routine appointments allowed this technique analysis afterwards.

The present study has some limitations. First, our findings are limited to the experience of a single surgical group. Second, a small sample size and retrospective analysis also collaborate to it intrinsic bias.

Our early outcomes appear favorable for this approach. It should be considered a possible approach for inguinoscrotal hernia repair whose presumable complications might not worth the risk of an extensive sac dissection. Further comparative studies may elucidate better its real benefits.

## CONCLUSION

This technique permits the management of large distal sacs avoiding clinical important injury to cord structures when repairing large inguinoscrotal hernias. It is reproducible and simple manner of dealing with inguinoscrotal hernia maintaining efficacy and lowering surgical time. 
